# Associations of adiponectin with individual European ancestry in African Americans: the Jackson Heart Study

**DOI:** 10.3389/fgene.2014.00022

**Published:** 2014-02-10

**Authors:** Aurelian Bidulescu, Shweta Choudhry, Solomon K. Musani, Sarah G. Buxbaum, Jiankang Liu, Charles N. Rotimi, James G. Wilson, Herman A. Taylor, Gary H. Gibbons

**Affiliations:** ^1^Department of Epidemiology and Biostatistics, Indiana University School of Public Health – BloomingtonBloomington, IN, USA; ^2^Department of Urology, University of CaliforniaSan Francisco, CA, USA; ^3^Jackson Heart Study, University of Mississippi Medical CenterJackson, MS, USA; ^4^Department of Health Sciences, Jackson State UniversityJackson, MS, USA; ^5^National Human Genome Research Institute, National Institutes of HealthBethesda, MD, USA; ^6^National Heart Lung and Blood Institute, National Institutes of HealthBethesda, MD, USA

**Keywords:** cohort study, adiponectin, individual European ancestry, minorities, African Americans, obesity, insulin resistance

## Abstract

**Background:** Compared with European Americans, African Americans (AAs) exhibit lower levels of the cardio-metabolically protective adiponectin even after accounting for adiposity measures. Because few studies have examined in AA the association between adiponectin and genetic admixture, a dense panel of ancestry informative markers (AIMs) was used to estimate the individual proportions of European ancestry (PEA) for the AAs enrolled in a large community-based cohort, the Jackson Heart Study (JHS). We tested the hypothesis that plasma adiponectin and PEA are directly associated and assessed the interaction with a series of cardio-metabolic risk factors.

**Methods:** Plasma specimens from 1439 JHS participants were analyzed by ELISA for adiponectin levels. Using pseudo-ancestral population genotype data from the HapMap Consortium, PEA was estimated with a panel of up to 1447 genome-wide preselected AIMs by a maximum likelihood approach. Interaction assessment, stepwise linear and cubic multivariable-adjusted regression models were used to analyze the cross-sectional association between adiponectin and PEA.

**Results:** Among the study participants (62% women; mean age 48 ± 12 years), the median (interquartile range) of PEA was 15.8 (9.3)%. Body mass index (BMI) (*p* = 0.04) and insulin resistance (*p* = 0.0001) modified the association between adiponectin and PEA. Adiponectin was directly and linearly associated with PEA (β = 0.62 ± 0.28, *p* = 0.03) among non-obese (*n* = 673) and insulin sensitive participants (*n* = 1141; β = 0.74 ± 0.23, *p* = 0.001), but not among those obese or with insulin resistance. No threshold point effect was detected for non-obese participants.

**Conclusions:** In a large AA population, the individual proportion of European ancestry was linearly and directly associated with plasma adiponectin among non-obese and non insulin-resistant participants, pointing to the interaction of genetic and metabolic factors influencing adiponectin levels.

## Introduction

Complex and common chronic diseases such as cardiovascular disease (CVD) which affect African Americans (AAs) disproportionally are likely caused by many genetic and environmental factors and their interactions (Go et al., 2014). Assessing associations between self-reported race/ethnicity with these diseases is often complicated due to heterogeneity within racial/ethnic groups (Burchard et al., 2003). The most abundant product of adipocytes, adiponectin is a global endophenotype for obesity and cardio-metabolic disease risk (Choi et al., 2004; Vettor et al., 2005; Wannamethee et al., 2007; Bidulescu et al., 2013a), and a potential mediator of the cardio-metabolic consequences of obesity (Ouchi et al., 2006). When compared to European Americans, AAs exhibit lower levels of the cardio-metabolically protective adipokine named adiponectin even after accounting for adiposity measures (Patel et al., 2006; Bidulescu et al., 2013b). The reason for this ethnic difference is still unclear.

African Americans in the United States are variably admixed with African and European ancestry (Parra et al., 2001). Due to this heterogeneity, genetic admixture analysis offers a unique opportunity for studying the role of genetic factors within a single, admixed population, controlling for socioeconomic and behavioral factors as well as comorbidities (Pritchard et al., 2000). Nevertheless, few studies have examined in AAs the association between adiponectin and individual ancestry estimates, as estimated with ancestry informative markers (AIMs), genetic loci showing alleles with large frequency differences between populations (Wassel Fyr et al., 2007). In addition, these studies used relatively few AIMs and small AA sample sizes.

We thus employed a relatively dense panel of AIMs to estimate the individual proportions of European ancestry (PEA) for the AAs enrolled in a large community-based cohort, the Jackson Heart Study (JHS) (Smith et al., 2004; Xu and Jin, 2008), in order to assess the association of this proportion with serum levels of the adiponectin. The hypothesis tested was that serum adiponectin and PEA are directly associated. For interaction assessment purposes, we also queried if obesity, insulin resistance and other cardio-metabolic measures modify this association.

## Materials and methods

### Study population

JHS is a single-site, prospective cohort study of the risk factors and causes of heart disease in adult AAs. A probability sample of 5301 AAs, aged 21–94 years, residing in a three county area surrounding the city of Jackson, MS, were recruited and examined at baseline (2000–2004) by certified technicians according to standardized protocols. Clinic visits and interviews occur approximately every 4 years. Annual follow-up interviews and cohort surveillance are ongoing. An overview of the JHS (Taylor, 2005) and details of the study design (Carpenter et al., 2004), recruitment protocol (Wyatt et al., 2003) and data collection methods (Carpenter et al., 2004) are published elsewhere.

The sample for this study included 1439 JHS participants (62% women; mean age 48 ± 12 years) with available DNA through the Candidate-Gene Association Resource (CARe) Study (Musunuru et al., 2010) and that had serum adiponectin measured (Bidulescu et al., 2011). Written consent was obtained from each participant at the inception of the study, and the study was approved by the participating JHS institutions: Jackson State University, Tougaloo College and the University of Mississippi Medical Center. The study protocol for the ascertainment of the adiponectin samples was approved by the Morehouse School of Medicine Institutional Review Board.

### Adiponectin measurements

Venous blood samples were withdrawn from each subject at baseline examination after more than 8 h of fasting as described elsewhere (Carpenter et al., 2004). Vials of serum were stored at the JHS central repository in Minneapolis, MN, at −80°C until assayed. Adiponectin concentration was measured as total adiponectin by an ELISA system (R&D Systems; Minneapolis, MN) (Bidulescu et al., 2011). The inter-assay coefficient of variation was 8.8%. No biological degradation has been described using stored specimens, indicating a high validity for our measurements.

### Proportion of european ancestry

In order to estimate ancestry we used a set of particularly informative DNA polymorphisms that have been termed AIMs due to their information content for distinguishing particular ancestral groups that correspond to continental populations. The proportion of European ancestry (as an estimate of the global proportion of individual European ancestry) was estimated for each individual using a panel of 1447 AIMs distributed across the genome. AIMs were selected using pseudo-ancestral population genotype data from the International HapMap Consortium to be informative for African vs. European ancestry (Smith et al., 2004; Xu and Jin, 2008). Ancestry proportions were estimated using a maximum likelihood approach in which the sum of the log-likelihood of individual ancestry from all the markers was maximized as a function of that person's ancestry, with a range between 0 and 1 (Chakraborty and Weiss, 1986; Long, 1991; Parra et al., 1998). If one considers an admixed population, K3, as the result from the genetic admixture of subjects from two ancestral populations, K1 and K2, then *s*1 and (1-*s*1) will represent the ancestry proportion from population K1 and K2, separately. If one represents *G*_*i*_ as the genotype for an admixed individual at the *i*th locus, then for *n* loci, likelihood can be defined as: *L*(*s1*) = ∏ i = 1 to *n Pr(G_i_*), where *Pr* is the actual proportion.

Instead of maximizing likelihood, it is computationally simple to maximize its natural logarithm: log_e_ [*L*(*s*1)] = ∑ i = 1 to *n* log_e_[*Pr(G_i_)*].

The MLE approach has been implemented in the program IAE3CI, which was kindly provided by Dr. Mark D. Shriver. The program requires the information of allele frequencies from each ancestral population and admixed subjects' genotyping data (Chakraborty et al., 1986; Hanis et al., 1986; Bonilla et al., 2004) We also compared our method with the algorithm for global ancestry estimation implemented in the other used programs. Tsai et al. (2005) evaluated the performances of three different methods for estimating global genetic ancestry: MLE, ADMIXMAP and STRUCTURE, through various simulated data sets and real data from Latino subjects participating in a genetic study of asthma. All three methods provided similar information on the accuracy of ancestral estimates. The Pearson's correlation coefficients, *r*, for ancestry estimates were >0.99 between MLE, ADMIXMAP, and STRUCTURE.

### CVD risk factors used as covariates

CVD risk factor information was ascertained during the JHS Exam 1 (2000–2004) through standard procedures and questionnaires. In all participants, the clinic visit included physical examinations, anthropometry, survey of medical history and current medication use and collection of blood and urine specimens for biological assessment. In-clinic standing height and weight were measured in lightweight examination clothing without shoes or constricting garments. We calculated body mass index (BMI) as weight in kilograms divided by height in meters squared (kg/m^2^). The average of two sitting blood pressure, measured at 1-min intervals after a 5-min silent rest, was used for analysis. Participants were considered current smokers if they smoked at the time of the baseline examination. Current alcohol use was defined as “yes” if they drank in the past 12 months. Lipid variables, fasting plasma glucose and fasting insulin were measured using standard laboratory techniques. Insulin resistance was calculated using the homeostasis model assessment for insulin resistance (HOMA-IR) (Matthews et al., 1985), and used as a continuous variable as well as a categorical variable. As a categorical variable, insulin resistance was defined as the upper quartile of the distribution as indicated by the fact that, considering the cardio-metabolic risk to establish the cut-off points for HOMA-IR, values between the 70th and the 75th percentile of HOMA-IR levels appears appropriate (Gayoso-Diz et al., 2013). Diabetes was defined as a fasting plasma glucose level of at least 7.0 mmol/l or if the subject was being treated with insulin or a hypoglycemic agent. Physical activity was assessed with a physical activity survey instrument administered by interview. The questionnaire assessed four different domains of physical activity (active living, work, home and garden, and sport and exercise indexes). A total score was composed as the sum of these domains (maximum of 24), with a higher score indicating a higher level of total physical activity (Dubbert et al., 2005). Obesity was defined by a BMI ≥ 30 kg/m^2^. C-reactive protein (CRP) was measured in duplicate by the immunoturbidimetric CRP-Latex assay from Kamiya Biomedical Company using a Hitachi 91l analyzer, according to the manufacturer's high-sensitivity protocol. The inter-assay coefficients of variation on control samples repeated in each assay were 4.5% at a CRP concentration of 0.45 mg/L and 4.4% at 1.56 mg/L.

### Statistical analysis

A cross-sectional analysis was performed. For each individual CVD risk factor, the Student unpaired *t*-test or the chi-square test was implemented in order to assess the significance of the difference between participants by sex.

Serum adiponectin levels were log-transformed in order to normalize their distribution and to meet assumptions about normality. Age- and sex-adjusted Pearson correlation coefficients between adiponectin, proportion of European ancestry and the main study covariates were calculated. Assessment of the interaction by sex, BMI, waist circumference, and insulin resistance were conducted using a log-likelihood testing. The effect measure modification potential of obesity and insulin resistance was assessed using interaction terms in the fully adjusted models. Multivariable linear regression models with a stepwise forward selection procedure were constructed with adiponectin as the dependent variable and PEA as the main independent variable. To confirm our findings, we have run a sensitivity analysis among participants without diabetes. For all analyses, the statistical significance was set at *P* ≤ 0.05 for main and interactive effect.

We also investigated the structure of the relationship between log-transformed serum adiponectin levels with proportion of European ancestry using cubic regression splines. In a multivariable-adjusted generalized additive model, we compared the deviance between models with and without proportion of ancestry. A non-linear relation was determined by a significant deviance and estimated degrees of freedom greater than 3.

All computations were performed using the SAS software version 9.2 (SAS® Institute Inc., Cary, North Carolina).

## Results

Our study sample comprised 897 women and 542 men. The average age was 48 ± 12 years. The serum adiponectin levels were statistically different between sexes: 5.3 ± 3.7 μg/mL in women and 3.8 ± 2.7 μg/mL in men. Among our study participants, the median (interquartile range) of PEA was 15.8 (9.3)%. There were statistically significant differences between sexes for adiponectin, BMI, waist circumference, triglycerides, HDL-cholesterol, fasting plasma glucose, HOMA-IR, type 2 diabetes, and CRP (Table 1) Among our study participants, 766 were obese, 209 were classified as having type 2 diabetes and 298 as insulin resistant participants (by HOMA-IR); 249 participants do not have insulin level measured, and thus did not have HOMA-IR estimated.

**Table 1:** **Descriptive characteristics (mean ± standard deviation, or percentage) of study participants (*N* = 1439) stratified by sex**.

	**Women (*N* = 897)**	**Men (*N* = 542)**	**Whole sample**
Age (years)	47 ± 12	49 ± 11	48 ± 12
Adiponectin (μg/mL)	5.3 ± 3.7	3.8 ± 2.7^*^	4.7 ± 3.4
PEA	17.0 ± 8.9	17.8 ± 8.1	17.3 ± 8.1
BMI (kg/m^2^; *n* = 1437^**^)	33.0 ± 7.4	29.5 ± 5.7^*^	31.7 ± 7.0
WC (cm; *n* = 1438)	99.3 ± 16.5	99.0 ± 14.2^*^	99.2 ± 15.7
TG (mg/dL; *n* = 1343)	97.0 ± 70.0	113.5 ± 118.5^*^	103.3 ± 91.7
HDL-C (mg/dL; 1344)	53.6 ± 13.8	45.5 ± 11.5^*^	50.5 ± 13.6
FG (mg/dL; *n* = 1344)	95.3 ± 27.5	96.7 ± 30.1^*^	95.8 ± 28.5
HOMA-IR (*n* = 1190)	3.7 ± 2.5	3.2 ± 2.0^*^	3.5 ± 2.3
Type 2 diabetes (%; *n* = 212)	16.3	12.1^*^	14.7
SBP (mm Hg; *n* = 1437)	121.5 ± 17.4	123.0 ± 16.2	122.1 ± 17.0
DBP (mm Hg; *n* = 1437)	77.7 ± 10.0	81.1 ± 10.5	79.0 ± 10.3
PA	8.8 ± 2.4	9.2 ± 2.4	8.9 ± 2.4
Alc. Drink. (% yes)	50	66	56
CRP (mg/dL)	6.1 ± 7.9	3.0 ± 5.5^*^	4.9 ± 7.2
Education (% > High School)	38	36	37

*PEA indicates proportion of European ancestry; BMI, body mass index; WC, waist circumference; TG, triglycerides; HDL-C, high-density lipoprotein cholesterol; FG, fasting plasma glucose; HOMA-IR, insulin resistance by homeostasis assessment model HOMA-IR; SBP, systolic blood pressure; DBP, diastolic blood pressure; PA, total physical activity score; Alc. Drink., Alcohol drinking in the past year; CRP, C-reactive protein*.

* *Statistical significant difference (t-test) by sex*.

** *Characteristics/parameters with less than the maximum number of 1439 participants are indicated in brackets (as the n number)*.

### Correlations between adiponectin proportion of european ancestry and other variables

Age- and sex-adjusted correlates of log-transformed adiponectin with the proportion of European ancestry and the CVD risk factors considered in our study are presented in Table 2. Adiponectin was significantly associated with the majority of covariates with the exception of systolic blood pressure, physical activity, and proportion of European ancestry (Table 2). Proportion of European ancestry was significantly associated with adiponectin and waist circumference, but not with HOMA-IR or BMI (Table 2). Thus, bivariate analyses among all our study participants revealed a statistically significant but weak correlation between PEA and adiponectin.

**Table 2:** **Age- and sex-adjusted Pearson correlation coefficients between adiponectin, proportion of European ancestry and covariates (*N* = 1439)**.

	Adiponectin^*^		**Proportion of European ancestry**	
	**Coefficient**	***p*-value**	**Coefficient**	***p*-value**
Adiponectin^*^	–	–	0.06	0.03
PEA	0.06	0.03	–	–
BMI (kg/m^2^)	−0.19	<0.0001	−0.05	0.11
WC (cm)	−0.25	<0.0001	−0.05	<0.0001
TG	−0.21	<0.0001	0.05	0.11
HDL-C	0.29	<0.0001	−0.02	0.52
FG	−0.22	<0.0001	−0.05	0.09
HOMA-IR	−0.33	<0.0001	−0.03	0.38
SBP	−0.03	0.31	−0.04	0.15
DBP	−0.04	0.19	−0.03	0.36
PA	−0.01	0.76	−0.06	0.04
CRP	−0.14	<0.0001	−0.01	0.66

*PEA indicates proportion of European ancestry; BMI, body mass index; WC, waist circumference; p, p-values; TG, triglycerides; HDL, high density lipoprotein cholesterol; FPG, fasting plasma glucose; HOMA-IR, insulin resistance (homeostasis model assessment); SBP, systolic blood pressure; DBP, diastolic blood pressure; PA, total physical activity score; CRP, C-reactive protein*.

* *Logarithmically-transformed values*.

### Multivariable-adjusted linear regression models of adiponectin and proportion of european ancestry

Body mass index (*p* = 0.04) and insulin resistance (*p* = 0.0001) were effect modifiers of the association PEA—adiponectin. On the contrary, sex (*p* = 0.64) and waist circumference (*p* = 0.83) were not found to be effect modifiers. Among non-obese individuals (*n* = 673), adiponectin was directly associated with PEA (β = 0.62 ± 0.28, *p* = 0.03), after adjustment for sex, waist circumference, systolic blood pressure, HOMA-IR, HDL-cholesterol and physical activity, as indicated by the stepwise procedure (Table 3).

**Table 3:** **Linear multivariable association between adiponectin and proportion of European ancestry**.

**By obesity status**	Non-obese (*N* = 673)^*^		Obese (*N* = 766)^*^	
	**β-coefficient**	***p*-value**	**β-coefficient**	***p*-value**
PEA	0.62	0.03	–	0.81
Age	–	0.61	0.005	0.04
Sex	−0.28	<0.0001	−0.32	<0.0001
WC	−0.01	0.01	–	0.31
TG	–	0.77	–	0.99
HDL	0.01	<0.0001	0.01	<0.0001
HOMA-IR	−0.09	<0.0001	−0.06	<0.0001
SBP	0.003	0.06	–	0.10
DBP	–	0.38	–	0.73
PA	−0.02	0.06	–	0.98
Alc. Drink.	–	0.33	–	0.91
CRP	–	0.29	−0.08	0.01
Education	–	0.22	–	0.56
**By insulin resist. status**	Without IR (*N* = 1141)^**^		With IR (*N* = 298)^***^	
	**β-coefficient**	***p*-value**	**β-coefficient**	***p*-value**
PEA	0.74	0.001	–	0.41
Age	0.005	0.006	0.06
Sex	−0.25	<0.0001	−0.24	0.001
BMI	0.01	0.003	–	0.41
WC	−0.01	<0.0001	–	0.28
TG	–	0.30	–	0.90
HDL	0.01	<0.0001	0.01	0.02
SBP	–	0.16	–	0.18
DBP	–	0.19	–	0.93
PA	–	0.10	–	0.61
Alc. Drink.	–	0.41	–	0.70
CRP	−0.08	0.01	–	0.37
Education	–	0.83	0.006	0.06

*PEA indicates proportion of European ancestry; BMI, body mass index; WC, waist circumference; TG, triglycerides; HDL, high density lipoprotein cholesterol; HOMA-IR, insulin resistance (homeostasis model assessment); SBP, systolic blood pressure; DBP, diastolic blood pressure; PA, total physical activity score; Alc. Drink., Alcohol drinking in the past year; CRP, C-reactive protein*.

* *R^2^ for the whole model = 0.22*.

** *R^2^ for the whole model = 0.10*.

*** *R^2^ for the whole model = 0.20*.

Among participants without insulin resistance (*n* = 1141), adiponectin was also directly associated with PEA (β = 0.74 ± 0.23, *p* = 0.001), after adjustment for age, sex, BMI, waist circumference, HDL-cholesterol and CRP, as indicated by the stepwise procedure (Table 3). In our sensitivity analyses, among participants without diabetes (*n* = 1106), there was a similar direct association between adiponectin and PEA (β = 0.52 ± 0.22, *p* = 0.02).

### Linear relationship between adiponectin and proportion of european ancestry

**Figure 1:**
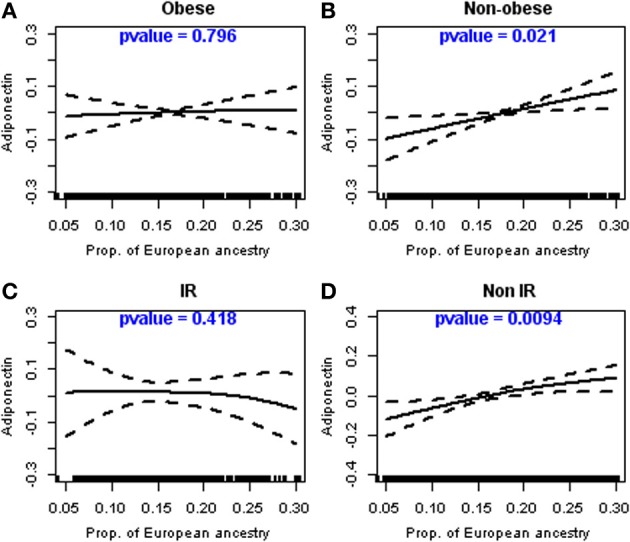
**Linear relationship between adiponectin and the proportion of European ancestry in non-obese and non-insulin resistant participants**. Legend: Relationship between the proportion of European ancestry and adiponectin levels (log-transformed), as described using cubic regression splines, stratified by obesity and insulin resistance levels; **(A)** Obese participants; **(B)** Non-Obese participants; **(C)** Participants with insulin resistance (IR); **(D)** Participants without insulin resistance (non-IR).

Figure 1 shows plots relating PEA to adiponectin after multivariable adjustment and stratification by obesity status (Figures 1A,B) and by insulin resistance status (Figures 1C,D). Test of deviation from linearity showed that log adiponectin was linearly and significantly (*p* = 0.021) related to PEA in the non-obese participants but not in the obese group (Figure 1B vs. Figure 1A). It also showed that adiponectin was non-linearly and borderline significantly (*p* = 0.091) related to PEA in a group of insulin resistant participants (results not shown), but after fitting a cubic regression smoother with greater than three degrees of freedom, it was obvious that the relation was linear but not significant (*p* = 0.418; Figure 1C). Adiponectin was on the other hand linearly and highly significantly (*p* = 0.0094) related to PEA among non-insulin resistant participants (Figure 1D). Overall the structure of relation between adiponectin to PEA seems to be linear, especially among non-obese participants.

## Discussion

### Principal findings

In a large community-based AA sample, the individual proportion of European ancestry was directly associated with serum adiponectin among non-obese and among insulin sensitive participants. There was not an association among obese participants or among those with insulin resistance. Across the entire distribution of adiponectin, there was a linear and statistically significant direct association between adiponectin and PEA among non-obese participants.

### In the context of previous literature

Few previous studies used estimates of individual ancestry to assess its association with CVD or other phenotypes (Wassel et al., 2009; Allison et al., 2010). A multivariable analysis in the Health ABC study showed a strong association between higher European ancestry and adiponectin levels (Wassel Fyr et al., 2007). The mean (standard deviation) of European ancestry among the 1241 AAs enrolled in that study was 22.3 (15.9)%, thus slightly higher compared to our investigation (Wassel Fyr et al., 2007). Estimates of European ancestry in our cohort were more consistent with other studies among AAs. For example, Parra and colleagues observed around 20% European ancestry for AA individuals from Pittsburgh, PA and around 12% in Charleston, SC (Parra et al., 1998).

African Americans in the United States are a heterogeneous group, variably admixed with African and European ancestry (Parra et al., 2001; Campbell et al., 2005). Because genetic-association studies are often performed in population samples of unrelated individuals to identify susceptibility loci for complex human traits, if subjects are sampled from two or more subpopulations for which the frequencies of marker alleles and traits differ, spurious associations may arise due to confounding by population substructure (Pritchard et al., 2000; Risch et al., 2002; Tang et al., 2005). Due to this heterogeneity, genetic admixture analysis offers a unique opportunity for studying the role of genetic factors within a single, admixed population, independent of social factors, and comorbidities. Ancestry informative markers, AIMs, are genetic loci showing alleles with large frequency differences between populations that can be used to estimate bio-geographical ancestry at the level of the population and individual. Ancestry estimates at both the subgroup and individual level can be directly instructive regarding the genetics of the phenotypes that differ qualitatively or in frequency between populations (Shriver et al., 2003). Specifically, an association between genetic ancestry and a disease phenotype within an admixed group such as AAs may be an indicator of genetic factors underlying differential expression among racial groups (Peralta et al., 2010).

Adiponectin, an adipose-specific protein, is generally negatively associated with adiposity, insulin sensitivity, and diabetes (Cnop et al., 2003; Duncan et al., 2004; Cote et al., 2005; Li et al., 2009). Nevertheless, it has been shown that ethnicity modifies the relationships of adiponectin and insulin resistance with obesity. For example, a recent study showed that BMI, insulin, and insulin resistance assessed through the homeostasis model assessment (HOMA) correlated significantly with adiponectin levels only in Caucasian women (Hulver et al., 2004). In another study, normal weight African women showed marginally lower adiponectin levels than their Caucasian counterparts (Schutte et al., 2007). Moreover, no differences in adiponectin were shown for overweight and obese African and Caucasian women (Schutte et al., 2007). These results indicate that what applies to other ethnic populations might not apply to the AA population, and that the association between adiponectin and insulin sensitivity needs to be clarified in the AA population.

The studies indicate that polymorphisms at the adiponectin locus are predictors of circulating adiponectin levels, insulin sensitivity, and atherosclerosis, highlighting the pivotal role of this adipokine in the modulation of metabolism and atherogenesis (Menzaghi et al., 2007). Several genetic loci are determinants of adiponectin levels, genetic determinants that taken together influence risk of type 2 diabetes and insulin resistance (Dastani et al., 2012). Variability at the adiponectin locus associated with obesity and other features of the insulin resistance syndrome has been studies intensively in the last years (Comuzzie et al., 2001; Menzaghi et al., 2007), but a limited number of studies have examined the role of heredity in the regulation of adiponectin among individuals of African heritage (Miljkovic-Gacic et al., 2007). Estimates of adiponectin heritability (adjusted for age, gender, and BMI) in two populations of African descent were 0.45 and 0.70 for the African American and Nigerian families, respectively, indicating a relatively high genetic determinant for adiponectin levels (Hicks et al., 2007). As our recent Mendelian randomization collaborative study indicated, by using an ADIPOQ genetic summary risk score, no evidence of an association between adiponectin-lowering alleles and insulin sensitivity or type 2 diabetes appears present (Yaghootkar et al., 2013). As our results suggest, measures of global individual ancestry obtained with relevant frequency genetic markers might be helpful for controlling spurious associations—relevant to this important adipokine—detected in population studies due to population stratification and admixture.

Our study showing a direct association between global proportion of European ancestry and adiponectin suggests that genetic factors are a significant source of inter-individual differences in circulating adiponectin among AAs. Moreover, as the effect modification (interaction) of obesity and insulin resistance status of the relation of adiponectin levels with PEA indicates, a complex interplay among specific genetic loci and non-genetic factors, which may both be associated with the overall admixture, might lead to the observed ethnic differences in cardio-metabolic risk. As we have shown (Bidulescu et al., 2013b), in JHS there are negative correlations between adiponectin and many of the CVD risk factors yet the women, who have higher adiponectin levels than men, also have higher CVD risk factors. If adiponectin acts as a compensatory mechanism to counteract these risk factors warrants additional studies.

An alternative mechanistic explanation for our findings may be that the association between ancestry and adiponectin is due to some non-genetic confounders, which were not characterized in our investigation. Although we adjusted for many of the known correlates of adiponectin levels, it is possible that other differences in medical treatment or additional socioeconomic and psychosocial factors for which we do not have information may underlie some of the observed associations.

### Strengths and limitations

Our study sample is localized to one geographical area and one ethnic group, so generalizability inherently cannot be inferred. As an “aggregate” measure, the global individual proportion of European ancestry might not capture the genetic influences of specific genetic regions. These caveats are compensated by the fact that we used a large number of AIMs, and were able to control for a large number of potential confounders in a large sample of AAs.

## Conclusion

Our study results point to the interaction of genetic and metabolic factors as variables associated with the lower levels of adiponectin in AAs. It warrants thus further exploration of the role ancestry plays in the complex relationship of adiponectin with cardio-metabolic risk factors.

### Conflict of interest statement

The authors declare that the research was conducted in the absence of any commercial or financial relationships that could be construed as a potential conflict of interest.
